# Supporting data for the MS identification of distinct transferrin glycopeptide glycoforms and citrullinated peptides associated with inflammation or autoimmunity

**DOI:** 10.1016/j.dib.2015.12.045

**Published:** 2016-01-11

**Authors:** A. Rosal-Vela, A. Barroso, E. Giménez, S. García-Rodríguez, V. Longobardo, J. Postigo, M. Iglesias, A. Lario, J. Merino, R. Merino, M. Zubiaur, V. Sanz-Nebot, J. Sancho

**Affiliations:** a Instituto de Parasitología y Biomedicina “López-Neyra” (IPBLN), CSIC, Armilla, Granada, Spain; b Departament de Química Analítica, Universitat de Barcelona, Spain; c Unidad de Proteómica, IPBLN, CSIC, Armilla, Granada, Spain; d Facultad de Medicina, Universidad de Cantabria, Santander, Spain; e Instituto de Biomedicina y Biotecnología de Cantabria, CSIC, Santander, Spain

**Keywords:** Arthritis, Inflammation, Protein species, Transferrin, Glycosylation, CD38, Citrullination

## Abstract

This data article presents the results of all the statistical analyses applied to the relative intensities of the detected 2D-DiGE protein spots for each of the 3 performed DiGE experiments. The data reveals specific subsets of protein spots with significant differences between WT and CD38-deficient mice with either Collagen-induced arthritis (CIA), or with chronic inflammation induced by CFA, or under steady-state conditions. This article also shows the MS data analyses that allowed the identification of the protein species which serve to discriminate the different experimental groups used in this study. Moreover, the article presents MS data on the citrullinated peptides linked to specific protein species that were generated in CIA^+^ or CFA-treated mice. Lastly, this data article provides MS data on the efficiency of the analyses of the transferrin (Tf) glycopeptide glycosylation pattern in spleen and serum from CIA^+^ mice and normal controls. The data supplied in this work is related to the research article entitled “identification of multiple transferrin species in spleen and serum from mice with collagen-induced arthritis which may reflect changes in transferrin glycosylation associated with disease activity: the role of CD38” [1]. All mass spectrometry data have been deposited to the ProteomeXchange Consortium via the PRIDE partner repository with identifiers PRIDE: PXD002644, PRIDE: PXD002643, PRIDE: PXD003183 and PRIDE: PXD003163.

**Specifications table**

**Table:** 

Subject area	*Biology*
More specific subject area	*Proteomics and glycoproteomics*
Type of data	*Tables, figures and raw data*
How data was acquired	Scanned 2D-DiGE images were analyzed using the DeCyder7.0 software (GE Healthcare) using the Differential In-gel Analysis (DIA) module to detect and normalize the protein spots. Protein relative abundance across all samples and statistical analyses were performed using the Biological Variation Analysis (BVA) module of the DeCyder software. *MS data for protein identification was acquired using a MALDI TOF/TOF UltrafleXtreme (Bruker), or a* 4800 MALDI-TOF/TOF Analyzer (AB SCIEX). *μLC–TOF–MS data for the analysis of the glycopeptides glycoforms of Tf was acquired with a 1200 series capillary liquid chromatography system (Agilent Technologies) coupled to a 6220 oa-TOF LC/MS mass spectrometer with an orthogonal G1385-44300 interface (Agilent Technologies).*
Data format	*Analyzed (excel files and word tables) and raw data*
Experimental factors	*Mice with Collagen-induced arthritis, or with chronic inflammation, or with no treatment. Protein extraction and/or purification from spleen or serum samples. CyDye labeling. 2-D gel electrophoresis.*
Experimental features	*Protein extracts from mice subjected to different experimental conditions were analyzed by 2D-DiGE, and protein species that differed in abundance were identified by MS/MS. PTMs such as citrullination of the identified proteins, or glycosylation of Tf species were further analyzed by MS.*
Data source location	*UB: Barcelona; UCO: Córdoba; IPBLN: Granada.*
Data accessibility	*Data is within this article. Data also available at the ProteomeXchange Consortium via the PRIDE partner repository, PRIDE: PXD002644, PRIDE: PXD002643, PRIDE: PXD003183 and PRIDE: PXD003163.*

**Value of the data**

• Application of μLC–TOF–MS for characterization of multiple glycopeptide glycoforms from mouse transferrin.
• Investigation of altered transferrin glycopeptide glycosylation patterns in inflammatory and/or autoimmune diseases.
• Mass spectrometry approach to identify new citrullinated peptides in mice with arthritis (CIA model).
• Properly described approach for 2D-DiGE analysis to identify protein species that differ in abundance due to certain pathologies.
• Basis for the study of altered protein species associated with inflammatory processes or arthritis in humans.

## 1 Data

**Fig. 1:**
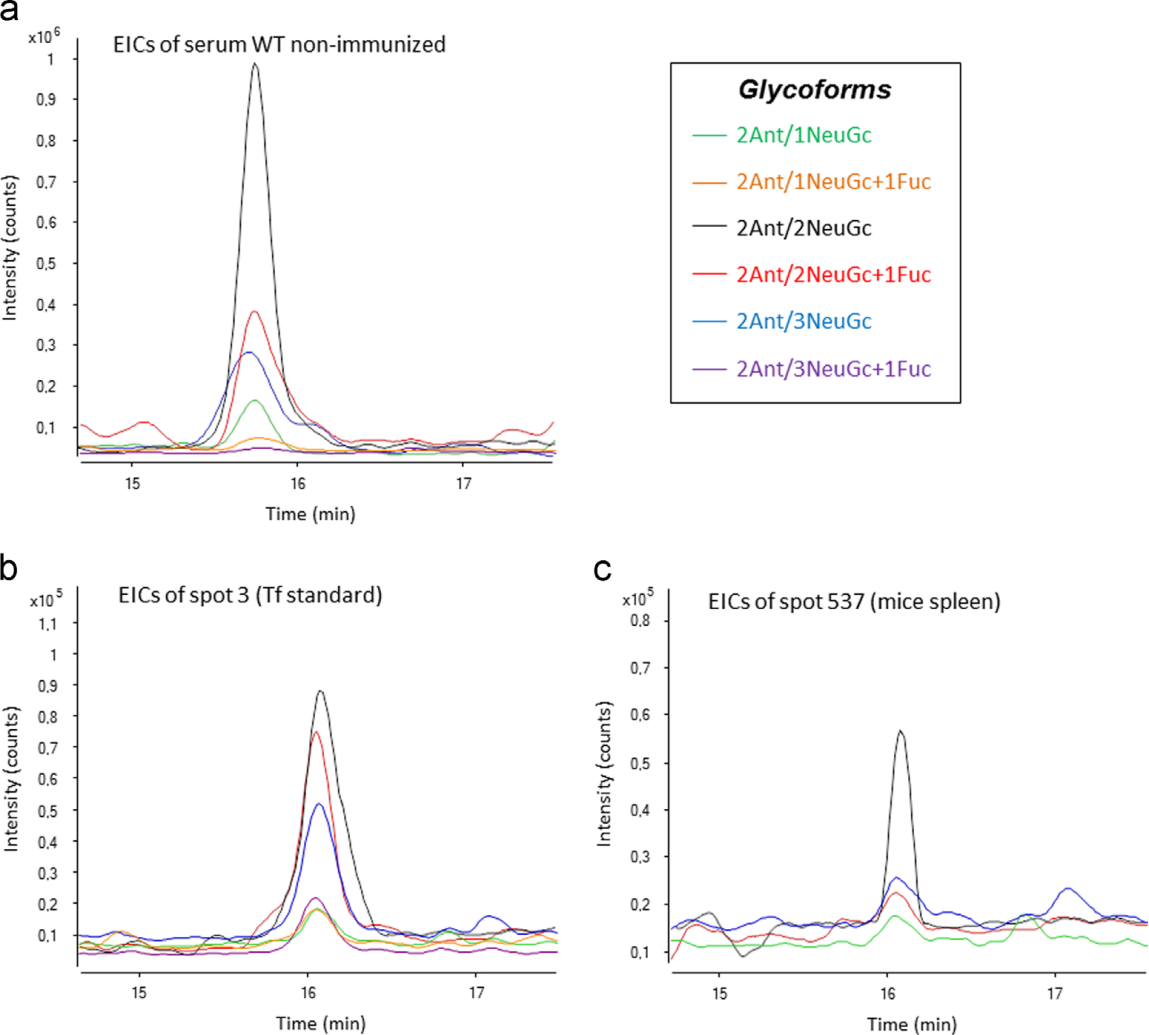
Extracted ion chromatograms (EICs) for the most abundant glycopeptide glycoforms of Tf isolated from (a) WT non-immunized serum, (b) Tf standard in a 2D gel (spot 3), and (c) Tf from a spleen extract in a 2D gel (spot 537 equivalent *pI* to spot 3) by µLC–TOF–MS.

Fig. 1 shows the extracted ion chromatograms (EICs) obtained by µLC–TOF–MS for the most abundant glycopeptide glycoforms of Tf isolated from WT non-immunized serum, Tf standard in a 2D gel, and Tf from a spleen extract in a 2D gel. Table 1, Table 2 in excel format show the list of the protein species identified by MS/MS, displaying the sequence of matched and fragmented peptides of a given protein. Table 3 shows the list of protein species identified by PMF. Table 4, Table 5, Table 6, Table 7, Table 8, Table 9, include the results of all the statistical analyses applied to the relative intensities of the detected 2D-DiGE protein spots for each of the 3 performed DiGE experiments. Table 10a, Table 10b shows the identities of the citrullinated peptides linked to specific protein species in CIA^+^, or CFA-treated mice. Table 11 shows the peptide coverage of mouse Tf standard digested with trypsin, and Table 12 shows the normalized peak area and %RSD of Tf glycopeptide glycoforms detected by µLC–TOF–MS in the spots of spleen protein extracts subjected to 2D electrophoretic separation and in-gel tryptic digestion.

## 2 Experimental design, materials and methods

### 2.1 Mice

WT mice were purchased from Harlan Ibérica (Barcelona, Spain). Mice deficient in CD38 (CD38-KO) were backcrossed onto the B6 background for more than 12 generations, as described previously [3]. All studies with live animals were approved by the IPBLN and Universidad de Cantabria Institutional Laboratory Animal Care and Use Committees.

### 2.2 Induction and assessment of arthritis

For the induction of CIA, 8–12 weeks-old male mice were immunized as previously described [4], [5].

### 2.3 Protein extraction from spleen preparations

Proteins were extracted from spleen by using the MicroRotofor Lysis Kit (for mammalian tissues and cells) (Bio-Rad, Ref #163-2141), following the manufacturer׳s instructions, which includes the use of mini-grinders for effective disruption of cells and tissues. The excess of salts and other contaminants were removed using the Bio-Rad׳s ReadyPrep 2-D cleanup kit. Samples were then resuspended in a DIGE-compatible buffer (7 M urea, 2 M thiourea, 4% CHAPS, 20 mM Tris, pH 8.5), quantified using the RC DC assay, and kept at −20 °C until further use.

### 2.4 Design of DiGE experiments

Unless otherwise indicated in each DiGE experiment conducted, four biological replicates of each condition were compared, comprising protein samples derived from four CD38-KO mice and four WT mice as previously described [1], [6].

### 2.5 DiGE labeling and two-dimensional gel electrophoresis

Samples were aliquoted at 45 μg, and the pooled internal standard was made with 23 μg of each of the sixteen test samples combined. The proteins were labeled with 400 pmol (in 1 μL of anhydrous DMF) of CyDye per 50 μg of protein as per the manufacturer׳s instructions (GE Healthcare). After labeling, the appropriate samples were combined for each gel. Each combined sample (~50 μL) was made up to 200 μL with Readyprep Rehydration/Sample buffer (8 M urea, 2% CHAPS, 50 mM dithiothreitol (DTT), 0.2% (w/v) Bio-Lyte® 3/10 ampholytes, and Bromophenol Blue (trace)).

2-DE was carried out using the Protean IEF cell and Criterion electrophoresis cell systems (Bio-Rad, Hercules, CA, USA) as previously described [7], with the following modifications: (1) First-dimension IPG strips (Bio-Rad: 11 cm, linear pH 3-10 gradient); (2) Active in-gel rehydration at 50 V, 12 h at 20 °C; (3) The IPG strips were focused in a one-step procedure, at 8000 V for a total of 35,000  Vh at 20 °C with a current limit of 50 μA/strip.

After electrophoresis, one of the gels was pre-scanned using the Typhoon 9400 variable mode imager at each of the appropriate CyDye excitation wavelengths (Cy3 (532 nm), Cy5 (633 nm), Cy2 (488 nm)), in order to determine the appropriate laser intensity for each CyDye. Thereafter, each of the analytical gels was scanned at this optimum laser intensity at a 2resolution of 100 μm. Gels were then fixed and stained with SYPRO Ruby (Bio-Rad) and re-scanned using the 488 nm laser. Scanned images were analyzed using the DeCyder7.0 software (GE Healthcare) using the Differential In-gel Analysis (DIA) module to detect and normalize the protein spots. Standard was used to normalize gels by calculating the standardized abundance of each spot, i.e., the ratio of either Cy3 or Cy5 signal to that of Cy2.

### 2.6 Protein identification by MALDI-TOF/TOF MS/MS

In-gel digestion of proteins has been described previously [8]. A set of protein spots were identified by MS/MS using a 4800 MALDI-TOF/TOF Analyzer (AB SCIEX) in automatic mode with the settings described previously [6]. Protein identification was assigned by peptide mass fingerprinting and confirmed by MS/MS analysis of at least three peptides in each sample. Mascot 2.0 search engine (Matrixscience) was used for protein identification running on GPS software (Applied Biosystems) against the SwissProt *Mus musculus* database (uniprot_sprot_26042011.fasta). The search setting allowed one missed cleavage with the selected trypsin enzyme, a MS/MS fragment tolerance of 0.2 Da and a precursor mass tolerance of 100 ppm.

Other spots were identified by MS/MS using a MALDI TOF/TOF UltrafleXtreme (Bruker) in manual mode as previously described [6]. Fragment selection criteria were a minimum S/N ratio of 15, a maximum number of peaks set at 200. For each precursor selected for MS/MS analysis, fragment mass values in the range from 13 Da to 4 Da below precursor mass were used to peptide identification.

Protein identification was assigned by peptide mass fingerprinting and confirmed by MS/MS analysis of 5 peptides. Mascot Server 2.4 (Matrixscience) and ProteinScape 3.1 (Bruker) were used for protein identification against the SwissProt *Mus musculus* database (SwissProt_2015_06.fasta and NCBInr_20150409.fasta). The search setting allowed two missed cleavage with the selected trypsin enzyme, fixed modification was cysteine carbamidomethylation and variable modification was methionine oxidation, a MS/MS fragment tolerance of 0.5 Da and a precursor mass tolerance of 50 ppm, unless otherwise indicated.

The MS spectra of the identified proteins were further examined in order to detect the presence of citrullinated proteins. Protein citrullination (o deimination) is the enzymatic conversion of peptidyl-arginine residues to peptidyl-citruline, mediated by the family of calcium-dependent peptidylarginine deiminases (PADs) [9]. The search setting for this PTM with MASCOT was performed as in the previous paragraph, including as variable modification the deamination of arginine, with the following considerations [10]: (a) for one citrullinated arginine, the peptide theoretical mass increase is 0.98 Da and the modified peptide, losing one amino group, becomes more acidic; (b) citrullinated arginine residues are not likely to be cleaved by trypsin, so that a minimum number of one missed cleavage must be specified; (c) a peptide that includes a C-terminal citrullinated arginine must be rejected; (d) citrullinated peptides generate an unusual isotopic mass cluster as compared with that of unmodified peptides.

### 2.7 μLC–TOF–MS

The µLC–TOF–MS experiments were performed in a 1200 series capillary liquid chromatography system coupled to a 6220 oa-TOF mass spectrometer with an orthogonal G1385–44300 interface (Agilent Technologies). LC and MS control, separation, data acquisition and processing were performed using MassHunter workstation software (Agilent Technologies). The oa-TOF mass spectrometer was tuned and calibrated following the manufacturer׳s instructions. Once a day, or even twice a day when required, a “Quick Tune” of the instrument was carried out in positive mode followed by a mass-axis calibration to ensure accurate mass assignments. In order to enhance detection sensitivity of glycopeptides, no internal recalibration was used [11]. MS measurement parameters were as described in a previous work [12]: capillary voltage 4000 V, drying gas (N_2_) temperature 200 °C,drying gasflow rate 4 L min^−1^,nebulizer gas (N_2_) 15 psig, fragmentor voltage 215 V, skimmer voltage 60 V, OCT 1 RF Vpp voltage 300 V. Data were collected in profile (continuum) at 1 spectrum s^−1^ (approx. 10,000 transients/spectrum) between m/z 100 and 3200, working in the highest resolution mode (4 GHz). For separation, a Zorbax 300SB-C18 column (3.5 m particle diameter, 300 A° pore diameter, 150 mM×0.3 mm LT×id, Agilent Technologies) was used. Experiments were performed at room temperature with gradient elution at a flow rate of 4 µL min^−1^. Eluting solvents were A: water with 0.1% (v/v) formic acid, and B: acetonitrile with 0.1% (v/v) formic acid. Solvents were degassed for 10 min by sonication before use. The optimum elution program was: solvent B from 10% to 60% (v/v) within 45 min as linear gradient, followed by cleaning and re-equilibration steps of B: 60% to 100% (v/v) (5 min), 100% (v/v) (10 min), 100% to 10% (v/v) (5 min) and 10% (v/v) (10 min). Before analysis, samples were filtered using a 0.22 µm polyvinylidene difluoride centrifugal filter (Ultrafree-MC, Millipore, Bedford, MA, USA) at 12,000 rpm for 4 min. Sample injection was performed with an autosampler refrigerated at 4 °C and the injection volume was 1 μL when analyzing Tf isolated from serum samples and digested with trypsin, and 5 μL when analyzing Tf in-gel digests.

### 2.8 μLC–TOF–MS data analysis

Prior to data analysis, a database with the exact monoisotopic mass of the different glycopeptide glycoforms of mouse Tf was created using Excel. To calculate the monoisotopic mass of each glycopeptide glycoform, it was necessary to calculate the elemental composition of all the glycopeptides taking into account the peptide and glycan contribution. First, the peptide sequence of mouse Tf was obtained from UniProt Knowledgebase (Q921l1), which also includes information about which cysteines and asparagines are involved in disulfide bonds and in *N*-glycosylation points, respectively. Afterwards, the theoretical sequence of each peptide and glycopeptide that would be obtained after tryptic digestion is obtained using the proteomic tool *PeptideMass* from the Expasy bioinformatics resource program. Subsequently, using the *ProtParam* tool from Expasy the elemental composition of the peptide sequence of the glycopeptide is obtained. Furthermore, the elemental composition of each glycan is calculated as the sum of the elemental composition of each monosaccharide that forms the glycan. *Ion source webpage* was used to obtain the elemental composition of each monosaccharide. Finally, the elemental composition of the peptide is added to obtain the molecular formula of each possible glycopeptides glycoform and thus, the monoisotopic mass with four decimals. Afterwards, the mass-to-charge values (m/z) for each glycopeptide glycoform are calculated up to a z value of 5 considering proton adducts (i.e. [M+H]^+^, [M+2H]^2+^, [M+3H]^3+^, [M+4H]^4+^ and [M+5H]^5+^)

Finally, the data analysis is carried out using the software MassHunter Qualitative (Agilent Technologies). All the previously calculated m/z values for each glycopeptide glycoform are extracted together to obtain an extracted ion chromatogram (EIC) of that glycopeptide specie, as can be observed in Fig. 1, which shows the EIC for some glycopeptide glycoforms in three different samples. If more than one of the extracted masses is detected in one chromatographic peak of the EIC, the presence of the corresponding glycopeptide glycoforms can be confirmed.

Table 1, Table 2 can be found in the online version of this article (.xlsx files). They show the list of protein species identified by MS/MS, displaying the sequence of matched and fragmented peptides of a given protein. Ion scores and confidence intervals of the fragmented peptides are also shown.

## Figures and Tables

**Table 1:** Protein spots from mouse spleen identified by MS/MS using the 4800 MALDI-TOF/TOF (AB Sciex).

**Spot number**^a^	**Identification**	**Accession**	**Accension number**^b^	**Protein score**	**Protein score CI%**^c^	**Pep.count**	**Total ion score**^d^	**Total %ion CI**	**MW (theorical)**^e^	**IEP (theorical)**^e^
533	Serotransferrin	TRFE_MOUSE	Q921I1	119	100	11	69	100.000	78840.5	6.94
537	Serotransferrin	TRFE_MOUSE	Q921I1	217	100	18	109	100.000	78840.5	6.94
539	Serotransferrin	TRFE_MOUSE	Q921I1	461	100	25	266	100.000	78840.5	6.94
614	Stress-induced-phosphoprotein 1	STIP1_MOUSE	Q60864	277	100	19	146	100	63,170	6.40
692	Fibrinogen beta chain	FIBB_MOUSE	Q8K0E8	800	100	25	578	100	55401.9	6.68
697	Catalase	CATA_MOUSE	P24270	179	100	10	131	100	60012.7	7.72
778	Alpha enolase	ENOA_MOUSE	P17182	891	100	27	594	100	47453.3	6.37
	Beta enolase	ENOB_MOUSE	P21550	250	100	6	224	100	47337.4	6.73
	Gamma enolase	ENOG_MOUSE	P17183	94	100	5	75	100	47609.1	4.99
906	Voltage-dependent anion selective channel protein 2	VDAC2_MOUSE	Q60930	179	100	11	95	100	32339.80	7
981	Proteasome subunit alpha type-1	PSA1_MOUSE	Q9R1P4	426	100	10	350	100.000	29812.9	6
982	Carbonic anhydrase 2	CAH2_MOUSE	P00920	478	100	17	295	100.000	29128.5	6.49
	Carbonic anhydrase 1	CAH1_MOUSE	P13634	203	100	7	155	100.000	28360.2	6.44
1001	Proteasome subunit alpha type-6	PSA6_MOUSE	Q9QUM9	335	100	10	264	100.000	27,811	6.34
	Pyridoxine-5′-phosphate oxidase	PNPO_MOUSE	Q91XF0	81	99.999	5	57	99.999	30437.1	8.46
1063	Flavin reductase	BLVRB_MOUSE	Q923D2	777	100	16	583	100.000	22297.4	6.49
1097	ATP synthase subunit d, mitochondrial	ATP5H_MOUSE	Q9DCX2	243	100	10	156	100.000	18794.6	5.52
1103	Ferritin light chain 1	FRIL1_MOUSE	P29391	585	100	14	435	100.000	20846.5	5.66
	Ferritin light chain 2	FRIL2_MOUSE	P49945	276	100	9	202	100.000	20886.9	6.39
1136	Peptidyl-prolyl cis-trans isomerase A	PPIA_MOUSE	P17742	340	100	10	249	100	18130.9000	8
1147	Actin-related protein 2/3 complex subunit 5-like protein	ARP5L_MOUSE	Q9D898	165	100	6	116	100	17026.80	6.32
1171	Nucleoside diphosphate kinase A	NDKA_MOUSE	P15532	654	100	13	518	100.000	17310.9	6.84
	Nucleoside diphosphate kinase B	NDKB_MOUSE	Q01768	330	100	7	278	100.000	17,466	6.97
1184	Protein S100-A9	S10A9_MOUSE	P31725	95	100	1	89	100	13,211	6.64
1313	Protein S100-A8	S10A8_MOUSE	P27005	146	100	3	119	100.000	10345.1	5.43


The sequence of matched and fragmented peptides of the identified proteins, plus the ion scores and confidence intervals of the fragmented peptides can be found in the online version of this article (Table 1, .xlsx file) as supplementary material.

a Spots are named as indicated on the 2-DE gel shown in Fig. 1 in Ref [1].

b UniProtKB/Swiss-Prot accession number, MASCOT protein score, protein score confidence interval (C.I. %), total ion score, and total ion score confidence interval (C.I. %) are reported for the combined search of MALDI-TOF/TOF MS and MS/MS data (GPS Software, Applied Biosystems).

c For protein scores, only confidence intervals above 99% were considered as statistically significant.

d For total ion scores, only confidence intervals above 95% were considered as statistically significant.

e Theoretical molecular weights and isoelectric points are given for each protein.

**Table 2:** Protein spots identified by MS/MS using the MALDI-TOF/TOF UltrafleXtreme (Bruker).

**Spot number**^a^	**Identification**	**Accession**	**Accession number**^b^	**Protein score**	**Sequence coverage (%)**	**No. of peptides**	**MW (theorical)**^c^	**IEP (theorical)**^c^
501	Aconitate hydratase, mitochondrial	ACON_MOUSE	Q99KI0	93.05	3.60	2	85,400	8.08
538	Serotransferrin	TRFE_MOUSE	Q921I1	96.65	4	3	78,841	6.94
539	Serotransferrin	TRFE_MOUSE	Q921I1	190.90	5.9	4	78,841	6.94
554	Far upstream element-binding protein 1	FUBP1_MOUSE	Q91WJ8	32.75	2.00	1	68,668	7.74
633	Fibrinogen alpha chain	FIBA_MOUSE	E9PV24	123.51	4.20	3	88,117	5.77
638	Heterogeneous nuclear ribonucleoprotein L	HNRPL_MOUSE	Q8R081	69.55	4.60	2	64,550	8.33
732	Coronin-1	CORO1A_MOUSE	gi|4895037	102.3	2.2	1	51,627	6.05
738	Vimentin	VIME_MOUSE	P20152	286.83	16.10	5	53,712	5.06
770	Protein disulfide-isomerase A6 precursor	PDIA6_MOUSE	gi|58037267	117.78	10.6	3	49,058	5.05
898	Actin, cytoplasmic 1	ACTB_MOUSE	P60710	177.47	9.10	2	42,052	5.29
	Beta-actin-like protein 2	ACTBL_MOUSE	Q8BFZ3	129.62	9.00	2	42,319	5.30
	F-actin-capping protein subunit alpha-1	CAZA1_MOUSE	P47753	92.04	9.80	2	33,090	5.34
972	Tropomyosin alpha-1 chain	TPM1_MOUSE	P58771	59.03	4.90	1	32,718	4.69
981	Proteasome subunit alpha type-1	PSA1_MOUSE	Q9R1P4	133.78	13.7	3	29,813	6.00
1001	Proteasome subunit alpha type-6	PSA6_MOUSE	Q9QUM9	177.79	16.3	4	27,811	6.34
1049	Proteasome subunit beta type-10	PSB10_MOUSE	O35955	109.4	8.80	3	29,330	6.40
	Growth factor receptor-bound protein 2	GRB2_MOUSE	Q60631	103.47	11.50	3	25,336	5.89
1103	Ferritin light chain 1	FRIL1_MOUSE	P29391	48.36	4.4	1	20,847	5.66
1150	E3 ubiquitin-protein ligase RNF181	RN181_MOUSE	Q9CY62	26.01	4.80	1	19,487	5.65
1173	Ubiquitin-conjugating enzyme E2 N	UBE2N_MOUSE	P61089	104.79	19.70	2	17,184	6.13
1302	N-acyl-aromatic-L-amino acid amidohydrolase (carboxylate-forming)	ACY3_MOUSE	Q91XE4	23.11	2.50	1	35,720	5.30

The sequence of matched and fragmented peptides plus the ion scores and confidence intervals of the fragmented peptides can be found in the online version of this article (Table 2, .xlsx file) as supplementary material.

a Spots are named as indicated on the 2-DE gel shown in Fig. 1 in Ref [1].

b UniProtKB/Swiss-Prot or NCBI accession number.

c Theoretical molecular weights and isoelectric points are given for each protein.

**Table 3:** Protein spots identified by PMF using the MALDI-TOF/TOF UltrafleXtreme (Bruker).

**Spot number**^a^	**Protein name**	**Accession number**^b^	**MW(theorical)**^c^	**IEP(theorical)**^c^	**Score**	**Expect**	**Sequence coverage**	**Queries matched**	**Queries searched**
572	Prelamin-A/C isoform A precursor (MS)	gi|162287370	74,478	6.54	77	0.0034	29	18	73
	Prelamin-A/C isoform C (MS)	gi|161760667	65,464	6.37	69	0.02	33	17	73
	Fibroblast growth factor 22 (MS)	gi|12963627	18,972	11.73	72	0.011	61	10	73
983	Mitochondrial peptide methionine sulfoxide reductase	Q9D6Y7	26,200	8.6	76	0.00042	34.3	7	37
1104	Low molecular weight phosphotyrosine protein phosphatase	Q9D358	18,636	6.30	58.5	2.40E−02	31.6	5	35

a Spots are named as indicated on the 2-DE gel shown in Fig. 1 in Ref [1].

b UniProtKB/Swiss-Prot or NCBI accession number.

c Theoretical molecular weights and isoelectric points are given for each protein.

**Table 4:** Spleen protein species that differ in abundance by 2-ANOVA-Mouse in Col II immunized CD38 KO mice versus B6 WT mice.

**DeCyder spot no.**	**Protein name**^a^	***P*****value (2-ANOVA-Mouse)**
**B6 WT:**		
**538**	Serotransferrin	3.99E−04
**633**	Fibrinogen alpha chain	6.11E−04
**692**	Fibrinogen beta chain	1.12E−03
**1097**	ATP synthase subunit d, mitochondrial	1.30E−03
**1302**	N-acyl-aromatic-L-amino acid	1.75E−03
	amidohydrolase (carboxylate-forming)	
**539**	Serotransferrin	3.53E−03
**533**	Serotransferrin	8.87E−03
**554**	Far upstream element-binding protein 1	9.53E−03
**537**	Serotransferrin	1.61E−02
**501**	Aconitate hydratase, mitochondrial	1.78E−02
**1171**	Nucleoside diphosphate kinase A	0.0195
	Nucleoside diphosphate kinase B	
**1103**	Ferritin light chain 1	2.06E−02
	Ferritin light chain 2	
**1300**	Not identified	0.023
**638**	Heterogeneous nuclear ribonucleoprotein L	2.89E−02
**572**	Prelamin-A/C isoform A precursor (PMF)	3.59E−02
	Prelamin-A/C isoform C (PMF)	
	Fibroblast growth factor 22 (PMF)	
**1313**	Protein S100-A8	0.05
**CD38 KO:**		
**898**	Actin, cytoplasmic 2	6.70E−03
	Beta-actin-like protein 2	
	F-actin-capping protein subunit alpha-1	
**982**	Carbonic anhydrase 2	8.98E−03
	Carbonic anhydrase 1	
**1044**	Not identified	0.0202
**981**	Proteasome subunit alpha type-1	2.39E−02

a Protein name according to UniProt, or to NCBI.

**Table 5:** Spleen protein species that differ in abundance by 2-ANOVA-Arthritis test in Col II-immunized CIA^+^ versus CIA^−^ mice.

**DeCyder spot no.**	**Protein name**^a^	***P*****value (2-ANOVA-Arthritis)**
**In CIA**^**+**^**:**		
**438**	Not identified	0.0162
**1150**	E3 ubiquitin-protein ligase RNF181	0.0414
		
**In CIA**^−^:		
**1157**	Not identified	0.021
**778**	Alpha-enolase	0.0365
	Beta-enolase	
	Gamma-enolase	

a Protein Name according to UniProt, or to NCBI.

**Table 6:** Spleen protein species that differ in abundance by 2-ANOVA-Interaction in two groups of Col.II-immunized mice (CD38 KO and B6 WT) with two conditions: CIA^+^ and CIA^−^).

**DeCyderspot no.**	**Protein name**^a^	***P*****value**(2-ANOVA-Mouse)	***P*****value**(2-ANOVA-Arthritis)	***P*****value**(2-ANOVA-Interaction)
**1302**	N-acyl-aromatic-L-amino acid	1.75E−03	0.535	1.27E−04
	amidohydrolase (carboxylate-forming)			
**532**	Not identified	0.065	0.585	5.29E−04
**538**	Serotransferrin	3.99E−04	0.255	8.40E−03
**982**	Carbonic anhydrase 2	8.98E−03	0.771	8.99E−03
	Carbonic anhydrase 1			
**1001**	Proteasome subunit alpha type-6	0.878	0.303	9.65E−03
	Pyridoxine-5׳-phosphate oxidase			
**983**	Mitochondrial peptide methionine	0.545	0.849	3.57E−02
	sulfoxide reductase (PMF)			
**1063**	Flavin reductase	0.707	0.779	0.0362
**537**	Serotransferrin	1.61E−02	0.306	4.07E−02

a Protein name according to UniProt, or to NCBI.

**Table 7:** Differences in spleen protein species abundance compared between Col II immunized CD38 KO mice (test group) versus Col II-immunized B6 WT mice (control group).

**DeCyder spot no.**	**Protein name**^a^	**Average ratio**^b^	***P*****value (*****t*****-test)**
		**Decreased abundance**	
**633**	Fibrinogen alpha chain	−1.35	1.67E−04
**692**	Fibrinogen beta chain	−1.19	4.53E−04
**1097**	ATP synthase subunit d, mitochondrial	−1.25	4.95E−04
**538**	Serotransferrin	−1.23	1.35E−03
**539**	Serotransferrin	−1.29	2.25E−03
**533**	Serotransferrin	−1.39	4.17E−03
**554**	Far upstream element-binding protein 1	−1.17	5.26E−03
**501**	Aconitate hydratase, mitochondrial	−1.17	9.81E−03
**1103**	Ferritin light chain 1	−1.37	0.0124
	Ferritin light chain 2		
**638**	Heterogeneous nuclear ribonucleoprotein L	−1.23	0.0142
**537**	Serotransferrin	−1.25	0.0208
**572**	Prelamin-A/C isoform A precursor (MS)	−1.18	0.0209
	Prelamin-A/C isoform C (MS)		
	Fibroblast growth factor 22 (MS)		
**1171**	Nucleoside diphosphate kinase A	−1.17	0.0258
	Nucleoside diphosphate kinase B		
**697**	Catalase	−1.16	0.0453
**536**	Not identified	−1.14	0.0475
**1300**	Not identified	−1.33	0.0493
			
		**Increased abundance**	
**898**	Actin, cytoplasmic 2	1.19	4.37E−03
	Beta-actin-like protein 2		
	F-actin-capping protein subunit alpha-1		
**1044**	Not identified	1.25	0.0163
**981**	Proteasome subunit alpha type-1	1.27	0.0194
**982**	Carbonic anhydrase 2	1.16	0.0457
	Carbonic anhydrase 1		

a Protein name according to UniProt, or to NCBI.

b Negative average ratios indicate decreased protein abundance, while positive ratios indicate increased protein abundance in CIA^+^ CD38 KO relative to that in CIA^+^ B6 WT mice.

**Table 8:** Chronic inflammation model. Differences in spleen protein species abundance compared between CFA/IFA-treated CD38 KO mice (test group) and CFA/IFA-treated B6 WT mice (control group).

**DeCyder spot no.**	Protein name^a^	Average ratio^b^	*P* value (*t*-test)
		**Decreased abundance**	
**1049**	Proteasome subunit beta type-10	−1.18	4.06E−03
	Growth factor receptor-bound protein 2		
**1330**	Not identified	−1.31	0.0106
**972**	Tropomyosin alpha-1 chain	−1.11	0.0163
**1173**	Ubiquitin-conjugating enzyme E2 N	−1.13	0.0217
**1103**	Ferritin light chain 1	−1.62	0.0343
	Ferritin light chain 2		
**738**	Vimentin	−1.22	0.0351
**1104**	Low molecular weight phosphotyrosine		
	protein phosphatase (PMF)	−1.28	0.0384
**732**	Coronin-1	−1.1	0.0434
		**Increased abundance**	
**1313**	Protein S100-A8	1.37	0.0236

a Protein name according to UniProt, or to NCBI.

b Negative average ratios indicate decreased protein abundance, while positive ratios indicate increased protein abundance in CFA/IFA-treated CD38 KO relative to that in CFA/IFA-treated B6 WT mice.

**Table 9:** Non-immunized control mice. Differences in spleen protein species abundance compared between non-immunized CD38 KO mice (test group) and non-immunized B6 WT mice (control group).

**DeCyder spot no.**	**Protein name**^a^	**Average ratio**^b^	***P*****value (*****t*****-test)**
		**Decreased abundance**	
**538**	Serotransferrin	−1.26	4.04E−03
**539**	Serotransferrin	−1.23	0.0185
**537**	Serotransferrin	−1.22	0.0221
**638**	Heterogeneous nuclear ribonucleoprotein L	−1.13	0.0266
**770**	Protein disulfide-isomerase A6	−1.11	0.0437
**532**	Not identified	−1.2	0.0477
		**Increased abundance**	
**1295**	Not identified	1.33	0.0162

a Protein name according to UniProt, or to NCBI.

b Negative average ratios indicate decreased protein abundance, while positive ratios indicate increased protein abundance in CFA/IFA-treated CD38 KO relative to that in CFA/IFA-treated B6 WT mice.

**Table 10a:** Citrullinated protein species and peptides^a^ detected in spleen from collagen-induced arthritis, or CFA-treated mice. TOF/TOF 4800.

**Spot number**	**Identification**	**Accesion**	**Accension number**	**Protein score**	**Protein score CI%**	**Pep. count**	**Total ion score**	**Total % ion CI**	**MW (theoretical)**	**IEP (theoretical)**

537	Serotransferrin	TRFE_MOUSE	Q921I1	172	100	20	107	100.00	78840.50	6.94
614	Stress-induced-phosphoprotein 1	STIP1_MOUSE	Q60864	238	100	27	145	100.00	63169.60	6.40
697	Catalase	CATA_MOUSE	Q8C6E3	174	100	15	131	100.00	60082.80	7.73
778	Alpha enolase	ENOA_MOUSE	P17182	940	100	33	675	100.00	47453.30	6.37
	Beta enolase	ENOB_MOUSE	P21550	260	100	12	224	100.00	47337.40	6.73
	Gamma enolase	ENOG_MOUSE	P17183	102	100	10	75	100.00	47609.10	4.99
981	Proteasome subunit alpha type-1	PSA1_MOUSE	Q9R1P4	415	100	14	350	100.00	29812.90	6.00
	Proteasome subunit alpha type	PSMA1_MOUSE	Q8BTU5	399	100	12	349	100.00	29732.80	5.78
1001	Proteasome subunit alpha type-6	PSA6_MOUSE	Q9QUM9	325	100	13	264	100.00	27811.00	6.34
	Pyridoxine-5׳-phosphate oxidase	PNPO_MOUSE	Q91XF0	81	99.999	5	57	100.00	30437.10	8.46
1103	Ferritin light chain 2	FRIL2_MOUSE	P49945	251	100	10	202	100.00	20886.90	6.39
1136	Peptidyl-prolyl cis–trans isomerase A	PPIA_MOUSE	P17742	320	100	11	249	100.00	18130.90	7.74
1171	Nucleoside diphosphate kinase A	NDKA_MOUSE	P15532	726	100	17	591	100.00	17310.90	6.84
	Nucleoside diphosphate kinase B	NDKB_MOUSE	Q01768	321	100	9	278	100.00	17466.00	6.97

a The sequence of matched citrullinated peptides of a given protein, and the positions of the deiminated arginines are shown in online version of this article as Supplementary material (Table 10, xlsx. File).

**Table 11:** Detected peptides in a tryptic digest of standard mTf analyzed by µLC–MS–TOF.

Detected peptides in mTf standard	
VPDK	✓
TVK	✓
WCAVSEHENTK	✓
CISFR	✓
DHMK	✓
TVLPPDGPR	✓
LACVK	✓
K	✓
TSYPDCIK	✓
AISASEADAMTLDGGWVYDA GLTPNNLKPVAAEFYGSVEH PQTYYYAVAVVK	X
K	✓
GTDFQLNQLEGK	✓
K	✓
SCHTGLGR	✓
SAGWVIPIGLLFCK	✓
LSEPR	✓
SPLEK	✓
AVSSFFSGSCVPCADPVAFP K	✓
LCQLCPGCGCSSTQPFFGYV GAFK	✓
CLK	✓
DGGGDVAFVK	✓
HTTIFEVLPEK	✓
ADR	✓
DQYELLCLDNTR	✓
KPVDQYEDCYLAR	✓
IPSHAVVAR	✓
K	✓
NNGK	X
EDLIWEILK	✓
VAQEHFGK	✓
GK	✓
SK	✓
DFQLFSSPLGK	✓
DLLFK	✓
DSAFGLLR	✓
VPPR	✓
MDYR	✓
LYLGHNYVTAIR	✓
NQQEGVCPEGSIDNSPVK	✓
WCALSHLER	✓
TK	✓
CDEWSIISEGK	✓
IECESAETTEDCIEK	✓
IVNGEADAMTLDGGHAYIAGQCGLVPVMAEYYESSNCAIPSQQGIFPK	✓
GYYAVAVVK	✓
ASDTSITWNNLK	✓
GK	✓
K	✓
SCHTGVDR	✓
TAGWNIPMGMLYNR	✓
INHCK	✓
FDEFFSQGCAPGYEK	✓
CAPNNK	✓
EEYNGYTGAFR	✓
CLVEK	✓
GDVAFVK	✓
HQTVLDNTEGK	✓
NPAEWAK	✓
NLK	✓
QEDFELLCPDGTR	✓
KPVK	✓
DFASCHLAQAPNHVVVSR	✓
K	✓
EK	✓
AAR	✓
VK	✓
AVLTSQETLFGGSDCTGNFC LFK	✓
STTK	✓
DLLFR	✓
DDTK	X
CFVK	✓
LPEGTTPEK	✓
YLGAEYMQSVGNMR	✓
K	✓
CSTSR	✓
LLEACTFHK	✓
H	✓
Total number of amino acids	678
Number of amino acids detected	618
Coverage (%)	91

**Table 12:** Normalized peak area and %RSD of Tf glycopeptide glycoforms detected in the spots of spleen protein extracts subjected to 2D electrophoretic separation and in-gel tryptic digestion.

Glycoforms	Spot 532		Spot 533		Spot 536		Spot 537		Spot 539	
*A*_norm_⁎	%RSD	*A*_norm_⁎	%RSD	*A*_norm_⁎	%RSD	*A*_norm_⁎^⁎^	%RSD	*A*_norm_⁎	%RSD
2Ant/1NeuGc	7.7	6.1	–	–	–	–	17.0	18.1	15.4	6.4
2Ant/1NeuGc1Fuc	5.5	1.2	–	–	–	–	–	–	–	–
2Ant/2NeuGc	54.7	3.7	56.9	7.1	54.9	7.9	76.5	5.5	137.8	0.8
2Ant/2NeuGc1Fuc	29.8	6.7	25.9	7.5	–	–	16.2	20.7	26.1	17.1
2Ant/3NeuGc	17.7	5.5	–	–	–	–	29.8	15.6	8.7	4.9
2Ant/3NeuGc1Fuc	2.6	3.3	–	–	–	–	–	–	–	–

⁎ *A*_norm_: normalized peak areas were calculated as: (Glycoform peak area/peptide 354–364 (CDEWSIISEGK) peak area)×100.

**Table 10b:** Citrullinated protein species and peptides^a^ detected in spleen from collagen-induced arthritis, or CFA-treated mice. TOF/TOF UltrafleXtreme.

**Spot number**	**Protein name**	**Accession**	**Accesion number**	**MW (theoretical)**	**IEP (theoretical)**	**Score**	**Sequence coverage (%)**	**Queries matched**	**Queries searched**

633	Fibrinogen alpha chain	FIBA_MOUSE	E9PV24	87.40	5.78	101.00	17.00	15	26
732	Coronin-1A	COR1A_MOUSE	O89053	51.00	6.04	83.40	36.00	16	75
898	F-actin-capping protein subunit alpha-1	CAZA1_MOUSE	P47753	32.90	5.34	77.90	43.00	12	65
972	Tropomyosin alpha-3 chain	TPM3_MOUSE	P21107	33.00	4.68	60.50	38.60	10	40

a The sequence of matched citrullinated peptides of a given protein, and the positions of the deiminated arginines are shown in online version of this article as Supplementary material (Table 10, .xlsx file).
